# Tacrolimus intra-patient variability is not associated with chronic active antibody mediated rejection

**DOI:** 10.1371/journal.pone.0196552

**Published:** 2018-05-10

**Authors:** Kasia A. Sablik, Marian C. Clahsen-van Groningen, Dennis A. Hesselink, Teun van Gelder, Michiel G. H. Betjes

**Affiliations:** 1 Department of Nephrology and Transplantation, Erasmus MC, University Medical Center, Rotterdam, The Netherlands; 2 Department of Pathology, Erasmus MC, University Medical Center, Rotterdam, The Netherlands; 3 Department of Hospital Pharmacy, Erasmus MC, University Medical Center, Rotterdam, The Netherlands; Children’s Hospital Boston, UNITED STATES

## Abstract

**Background:**

Chronic active antibody mediated rejection (c-aABMR) is a major cause of long-term kidney allograft loss. It is hypothesized that frequent sub-therapeutic exposure to immunosuppressive drugs, in particular tacrolimus (Tac), is a risk factor for the development of c-aABMR. The intra-patient variability (IPV) in Tac exposure may serve as a substitute biomarker for underexposure and/or non-adherence. In this study, the association between Tac IPV and the development of c-aABMR was investigated.

**Methods:**

We retrospectively included 59 patients diagnosed with c-aABMR and compared them to 189 control patients matched for age, year of transplantation and type of kidney donor. The Tac IPV was calculated from pre-dose tacrolimus concentrations measured over a 3 year period preceding the diagnosis of c-aABMR. The mean Tac predose concentrations (C_0_), Tac IPV, renal allograft function and graft survival were compared between the groups.

**Results:**

Tac IPV was 24.4% for the cases *versus* 23.6% for the controls (p = 0.47). The mean Tac C_0_ was comparable for the cases (5.8 ng/mL) and control patients (6.1 ng/mL, p = 0.08). Only in the c-aABMR group a significant decline in both mean Tac C_0_ and allograft function over the timespan of 3 years was observed (p = 0.03 and p<0.001). Additionally, in the group of c-aABMR patients a high IPV was associated with inferior graft survival (p = 0.03).

**Conclusions:**

A high Tac IPV *per se* does not predispose to the development of c-aABMR but is associated with inferior graft survival once c-aABMR is diagnosed.

## Introduction

Despite a significant improvement in short-term kidney allograft survival in the past decade, long-term kidney allograft survival has remained relatively unchanged[1]. Chronic active antibody mediated rejection (c-aABMR) contributes substantially to these disappointing long-term transplantation outcomes[2, 3]. c-aABMR is believed to be the result of recurrent endothelial activation by pre-existing or de novo anti-HLA antibodies leading to numerous pathological abnormalities[4, 5]. It has been hypothesized that the development of c-aABMR is partially attributable to poor adherence or inadequate maintenance immunosuppression[3, 6].

In most centers, tacrolimus (Tac) is the cornerstone of the maintenance immunosuppressive regimen after renal transplantation[7]. Tac is a drug that requires frequent predose concentration monitoring to maintain therapeutic exposure[8]. It has a narrow therapeutic window and displays considerable intra-patient variability (IPV). The Tac IPV is defined as the fluctuation in Tac concentrations within an individual patient over a certain period of time[9]. These fluctuations in Tac exposure may result in periodic excessive or insufficient exposure, possibly leading to (nephro)toxicity or acute rejection. Many studies have reported an association between a high Tac IPV and inferior graft outcomes[10]. Patients with more variability in Tac exposure were more likely to develop donor-specific anti-HLA antibodies, lose their grafts and develop chronic histological lesions[3, 11–17]. In these studies, allograft failure was often defined as a composite endpoint which included the diagnosis of c-aABMR[3, 6, 11, 12, 15, 18]. However, the association between Tac IPV and the development of c-aABMR has never been analyzed separately. In this study, the association between Tac IPV and the risk of c-aABMR was investigated.

## Subjects and methods

### Study population

For this retrospective case-control study, all kidney transplant recipients transplanted in our center between 2000 and 2013 were eligible. The main inclusion criteria was the use of Tac as maintenance immunosuppression for both cases and controls. The year 2000 was chosen because then Tac became the CNI of choice in our center.

The standard immunosuppressive regimen did not include induction therapy before the year 2006–2007. Thereafter, patients received induction therapy with basiliximab and were set on a triple immunosuppressive regimen consisting of tacrolimus, mycophenolate mofetil and prednisolone after transplantation. In the first year after transplantation prednisolone was slowly tapered to 0 after 3 months. As per local protocol, the administration of Tac was slowly tapered after the first 6 months and Tac C_0_ were aimed at levels between 5–7 ng/ml. There were no other inclusion criteria regarding the use of other (maintenance) immunosuppressive drugs. This retrospective study was reviewed and approved by the Institutional Ethics Committee from the Erasmus MC, Rotterdam, The Netherlands. Due to the retrospective nature of the study no informed consent was needed.

Patients diagnosed with histologically-proven (suspicious) c-aABMR were defined as cases. The cases were selected from the pathology database at our center and were diagnosed after a for-cause biopsy. The diagnosis of c-aABMR was made at time of biopsy by an experienced renal pathologist based on the current Banff classification[19–21]. Patients were excluded if insufficient data was available (see data collection).

As a control group we selected kidney transplant recipients that showed no evidence of c-aABMR. All patients in the control group have had no clinical suspicion of c-aABMR until point of inclusion. Additionally, if present, all previous biopsy results have been screened for signs of (suspicious) c-aABMR. Controls were matched for age, year of transplantation, type of kidney donor (deceased *versus* living) and a minimal graft survival which resembled the cases’ time to c-aABMR diagnosis. The matching process was performed by coding all patients, both cases and controls, for the aforementioned factors. By means of an algorithm patients were found to be a positive match if all 4 factors were alike. The matches were selected from the transplantation database of our center. For every single c-aABMR case all possible matches were included.

### Data collection

Demographic and baseline transplantation characteristics were collected for all patients. These characteristics were retrieved from the local transplantation database and complete for more than 90%. In addition, data on absolute (*i*.*e*. nondose-corrected) tacrolimus whole-blood, pre-dose concentrations were collected. Only Tac C_0_ measured after month 6 after transplantation were included, to allow for stabilization of drug dosing[11, 12]. Furthermore, only Tac C_0_ sampled during outpatient clinic visits were included to minimize bias from measurements made during hospitalization.

Tac C_0_ were collected over a period of 3 years. A minimum of 8 out-patient clinical measurements of Tac C_0_ over a minimal time period of 2 years was necessary per individual for an adequate calculation of Tac IPV.

The mean Tac C_0_ exposure over the 3 years was calculated as well as the Tac C_0_ exposure per year. The Tac IPV of the cases was calculated in the 3 year time period prior to c-aABMR diagnosis (endpoint, t_0_). The IPV of the controls was calculated over a similar time period, dependent on the matched cases’ time to endpoint (t_0_). The Tac IPV of interest for each patient was calculated as follows:

$$\{[\left(X_{mean}-X_{1})+(X_{mean}-X_{2})\ldots +(X_{mean}-X_{n}\right)]÷n\}÷X_{mean}\times 100$$

Formula 1. Formula used for calculating the intra-patient variability often also referred to as mean absolute deviation (MAD)[9, 11]. X_mean_ is the mean Tac C_0_ of all available samples in the 3 years prior to c-aABMR diagnosis. X_1_ represents the first available Tac C_0_ measurement, X_2_ the second…, and so on.

Furthermore, estimated glomerular filtration rate (eGFR, MDRD) measurements were collected at routine out-patient clinic controls[22]. Data was collected at the time of endpoint (t_0_) and 1 (t_-1_), 2 (t_-2_), and 3 (t_-3_) years prior to the histological diagnosis of c-aABMR (or matched controls endpoint). Data on allograft function was complete up to 90%.

### Outcome

The association between Tac IPV and the development of c-aABMR was the primary outcome of interest. Secondary outcomes were allograft function, the mean Tac C_0_ exposure and its change over time for both the cases and controls. Additionally, the change in mean Tac C_0_ exposure per year and its relation to allograft function was assessed. Potential confounders such as age, sex, time to diagnosis and type of transplantation were analyzed as possible contributing factors to high Tac IPV in patients with c-aABMR.

### Statistical analysis

The baseline characteristics of both cases and controls are reported using summary statistics and frequency tables for continuous and categorical variables. Differences between cases and controls were analyzed by the Chi-square test for categorical variables and with independent and paired sample t-test test for continuous variables, as appropriate. Additionally, several clinical characteristics such as age, gender, type of transplantation, mismatch and time to c-aABMR diagnosis were analyzed for association with Tac IPV in the c-aABMR cases. The groups were divided based on the characteristic and IPV percentages were compared by means of independent sample t-test. Linear regression was used to examine the correlation between allograft function and mean Tac C_0_. Overall graft survival was assessed by Kaplan-Meier survival analysis with log-rank statistics for difference. The software IBM SPSS statistics 21 was used to perform the statistical analysis. Variables were considered statistically significant with a two-tailed P-value of <0.05.

## Results

### Patient characteristics

Two hundred and forty-eight renal transplant patients were identified for this study and included for analysis. Fifty-nine patients were considered cases and the remaining 189 were matched controls. The characteristics of cases and controls are displayed in Table 1. The median time from kidney transplantation to c-aABMR diagnosis (t_0_, endpoint) was 6.1 (IQR 3.5–8.3) years. The follow-up for both cases and controls was 3 years. Matching was achieved successfully as there were no differences between cases and controls with regard to age at transplantation, donor type and year of transplantation. More importantly, no statistically significant differences were found in mean eGFR (48 ml/min/1.73m^2^ cases vs. 50 ml/min/1.73m^2^ controls, p = 0.55) and mean Tac C_0_ (6.1 ng/ml vs. 6.1 ng/ml, p = 0.98) at starting point (t_-3_) which was 3 years prior to c-aABMR diagnosis or matched controls endpoint. There was however a significant difference in gender with significantly more male patients in the c-aABMR cases.

**Table 1:** Demographic and clinical characteristics.

		Cases (n = 59)	Controls (n = 189)	p-value
**Age,** *years*, *mean (sd)*		49 (±13)	52 (±13)	0.23
**Gender,** *n (%)*	Female	21 (36)	39 (21)	0.02
	Male	38 (64)	150 (79)	
**Donor type,** *n (%)*	Deceased donor	19 (32)	41 (22)	0.10
	Living donor	40 (68)	148 (78)	
**Donor age,** *years*, *mean (sd)*		51 (±12)	51 (±13)	0.71
**Previous Transplantation,** *n (%)*	Yes	12 (20)	24 (13)	0.15
	No	47 (80)	165 (87)	
**Time of Transplantation,** *year*		2000–2012	2000–2012	
**Time to diagnosis,** *years*, *median (IQR)*		6.1 (3.5–8.3)	-	
**Total HLA mismatch,** *median (IQR)*		3 (2–5)	3 (3–5)	0.70
**PRA**^a^ **current,** *mean (range)*		1.5 (0–8)	3.2 (0–96)	0.44
**PRA peak,** *mean (range)*		9.5 (0–62)	6.6 (0–80)	0.49
**Previous BPAR**^b^, *n (%)*	Yes	15 (25)	36 (19)	0.30
	No	44 (75)	153 (81)	
**eGFR at t_-3_,** *ml/min/1*.*73m*^*2*^, *mean (sd)*		48 (±12)	50 (±15)	0.55
**Trough-level at t_-3_,** *ng/mL*, *mean (sd)*		6.1 (±1.9)	6.1 (±1.7)	0.98
**C_0_ measurements,** *n*, *mean (range)*		17 (8–33)	15 (8–44)	0.01
**Primary kidney disease,** *n (%)*	Diabetic	8 (13.6)	36 (19)	
	Nephropathy			
	Hypertensive	10 (16.9)	44 (23.3)	
	Nephropathy			
	IgA Nephropathy	3 (5.1)	12 (6.3)	
	Polycystic Kidney	10 (16.9)	24 (12.7)	
	Disease			
	FSGS^c^	3 (5.1)	8 (4.2)	
	Obstructive	1 (1.7)	7 (3.7)	
	Nephropathy			
	Unknown	3 (5.1)	11 (5.8)	
	Other	21 (35.6)	47 (25)	

^a^ PRA, Panel Reactive Antibody;

^b^ BPAR, biopsy proven acute rejection (incl. borderline changes);

^c^ FSGS, Focal Segmental Glomerulosclerosis

### Tacrolimus intra-patient variability and mean tacrolimus predose concentrations

For the calculation of Tac IPV on average 17 (8–33) Tac C_0_ measurements were available for the cases and 15 (8–44) measurements for the controls. The number of available Tac C_0_ measurements was significantly higher for the cases (p = 0.01). This slight but significant difference could be explained by the increased number of out-patient clinic visits (related to impairment of renal function or the appearance of proteinuria) prior to the for-cause biopsy, in the cases.

The mean Tac IPV was 24.4% (range: 12.0%-48.3%) in the cases and 23.6% (range: 9.7%-46.3%) in the controls and showed no statistically significant difference (p = 0.47). We were unable to identify any specific clinical characteristics, such as age, gender, type of transplantation (living or deceased) and time to c-aABMR diagnosis to be associated with a significant difference in IPV (Table 2).

**Table 2:** Clinical characteristics associated with IPV in c-aABMR cases.

Clinical characteristic		Tac IPV, %, mean (sd)	p-value
**Age**	<50yrs (n = 29) >50yrs (n = 30)	22.8% (±7.4) 25.9% (±7.4)	0.12
**Gender**	Male (n = 38) Female (n = 21)	24.9% (±7.7) 23.5% (±7.2)	0.51
**Type of transplantation**	PM (n = 19) Living (n = 40)	24.4% (±5.9) 24.4% (±8.2)	0.99
**Mismatch**	Mismatch<3 (n = 31) Mismatch>3 (n = 26)	24.1% (±7.1) 24.8% (±8.3)	0.72
**Time to c-aABMR**	<2250 days (n = 30) >2250 days (n = 29)	25.4% (±7.3) 23.3% (±7.7)	0.28

Similarly, there was no significant difference in mean Tac C_0_ over the total 3 year period (t_-3_-t_0_). The cases had a mean Tac C_0_ of 5.8 ng/mL [range: 3.3 ng/mL-8.5 ng/mL] *versus* a mean Tac C_0_ of 6.1 ng/mL [range: 3.6 ng/mL-10.0 ng/mL] for the controls (p = 0.08).

**Fig 1:**
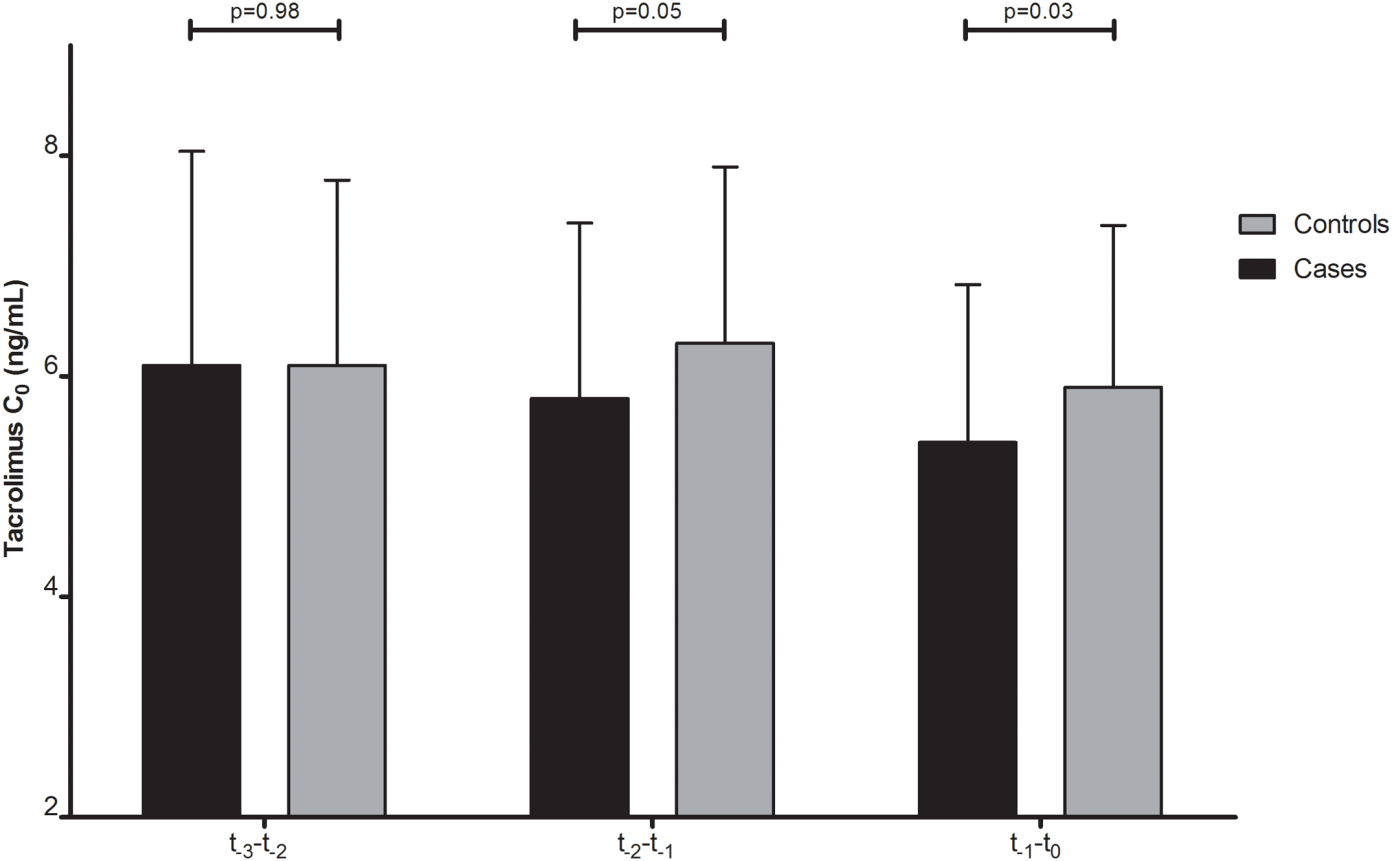
The tacrolimus (Tac) predose concentrations (C_0_) spread over the 3 years prior to c-aABMR diagnosis/matched controls endpoints (t_0_) for both c-aABMR patients (cases) and controls. Tac C_0_ (t_-3_-t_-2_) was 6.1 (±1.94) ng/mL for both cases and controls. Tac C_0_ (t_-2_-t_-1)_ was 5.8 (±1.59) ng/mL for the cases and 6.3 (±1.60) ng/mL for the controls. Tac C_0_ (t_-1_-t_-0_) was 5.4 (±1.43) ng/mL for the cases and 5.9 (±1.47) ng/mL for the controls.

There was however a significantly declining trend noted in mean Tac C_0_ during the 3 years preceding the diagnosis of c-aABMR among cases but not controls (Fig 1) (p = 0.02 for cases *versus* p = 0.18 for controls). When mean Tac C_0_ was analyzed per year, patients who developed c-aABMR had a mean Tac C_0_ of 6.1 ng/mL between t_-3_-t_-2_, which fell to 5.8 (t_-2_-t_-1_) and 5.4 ng/mL (t_-1_-t_-0_) in the years thereafter. Controls had a similar mean Tac C_0_ to the cases between t_-3_-t_-2_ (6.1 ng/mL; p = 0.98) which in the years thereafter showed no decline (6.3 ng/mL (t_-2_-t_-1_) and 5.9 ng/mL (t_-1_-t_-0_)). The difference in mean Tac C_0_ in the year prior to endpoint (t_-1_-t_-0_) between the cases and controls was significant at a P-value of 0.03.

### Allograft function

**Fig 2:**
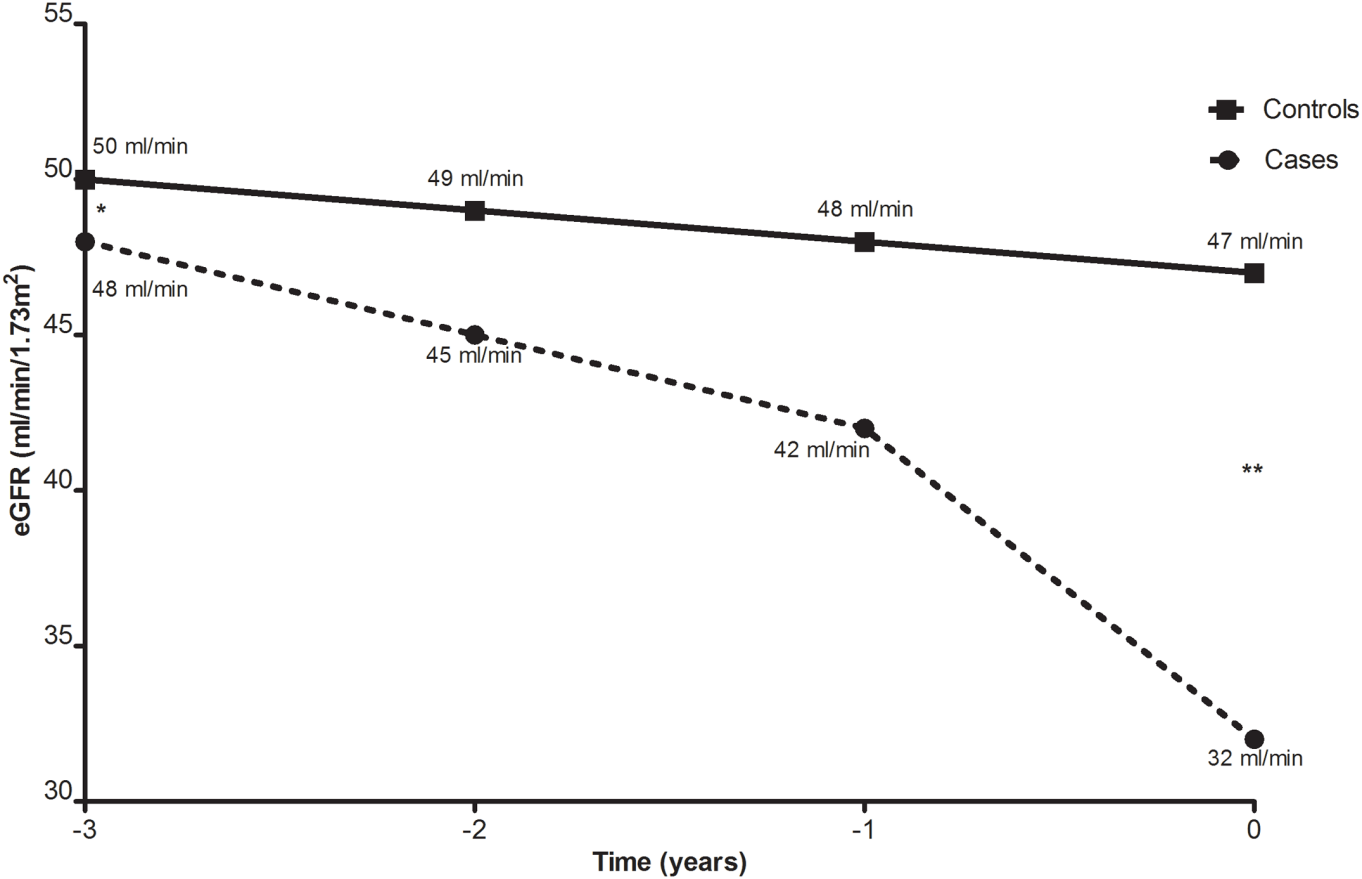
Allograft function of both patients with c-aABMR (cases) and controls. *t_-3_, p = 0.55; **t_0_, p<0.001.

Cases and controls showed a similar allograft function at start of analysis (t_-3_) of 48 ml/min/1.73m^2^ and 50 ml/min/1.73m^2^, respectively (p = 0.55). In the following years the cases had a substantial decline in allograft function. The average eGFR of the cases deteriorated from 48 ml/min/1.73m^2^ 3 years before the diagnosis of c-aABMR to 45 (t_-2_), 42 (t_-1_), and 32 ml/min/1.73m^2^ (t_0_; Fig 2). The controls on the other hand had a stable decline in allograft function of only 1 ml/min/1.73m^2^ per year. The average eGFR of the controls decreased from 50 (t_-3_) to 49 (t_-2_), to 48 (t_-1_), and to 47 ml/min/1.73m^2^ (t_0_; Fig 2). The allograft function of cases and controls did not differ significantly in the first 2 years of follow up (p = 0.55 at t_-3_ and p = 0.07 at t_-2_). However, in the 2 years thereafter the cases had a significantly inferior allograft function compared to the controls (p = 0.005 at t_-1_ and p<0.001 at t_0_).

**Fig 3:**
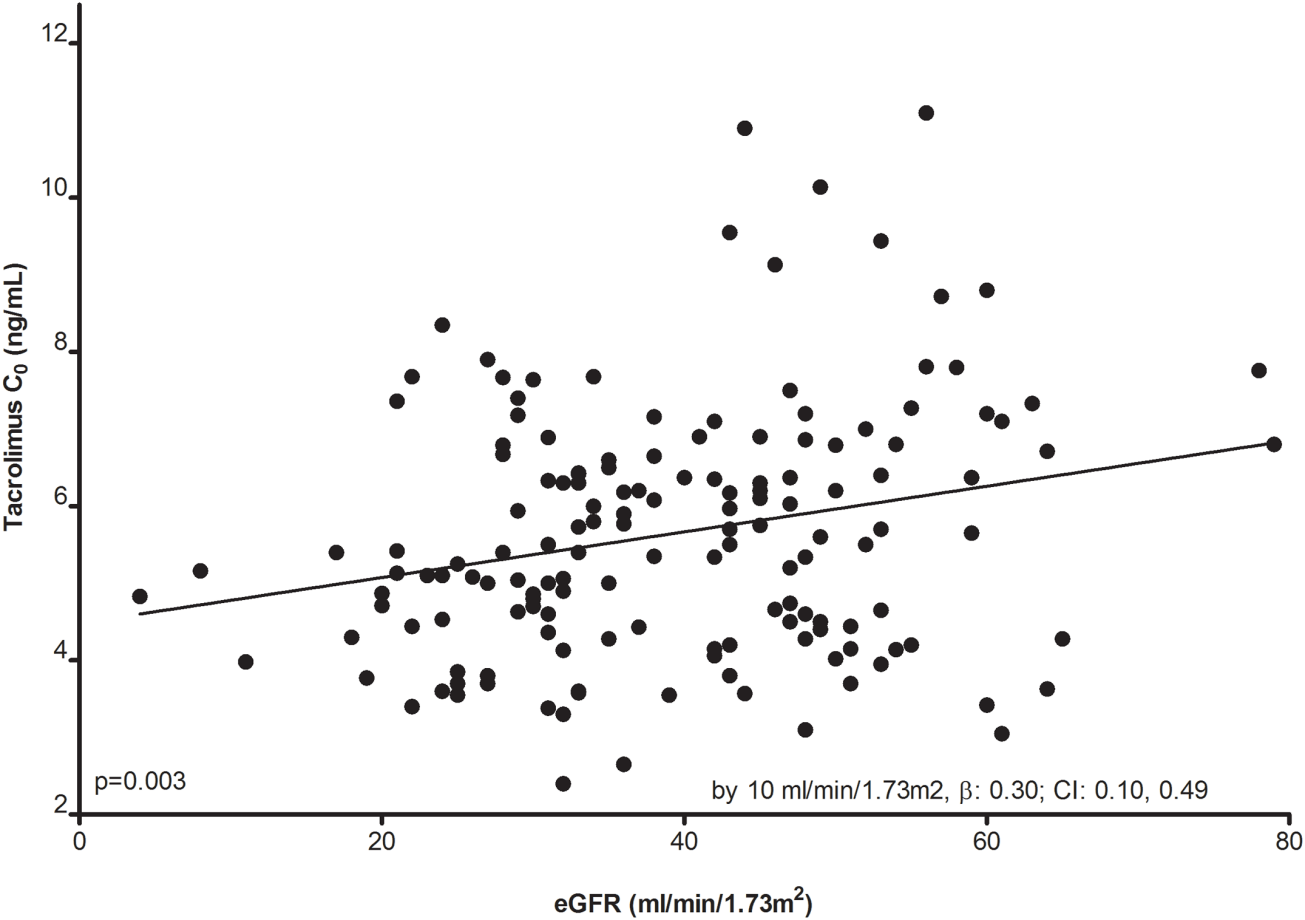
Association between tacrolimus (Tac) predose concentrations (C_0_) and allograft function for the c-aABMR patients (cases).

Linear regression analysis showed a positive association between increasing Tac C_0_ and a better allograft function (by 10 ml/min/1.73m^2^, β: 0.30; CI: 0.10, 0.49; P = 0.003, Fig 3). This correlation was not present for the controls (p = 0.49).

### Allograft survival

**Fig 4:**
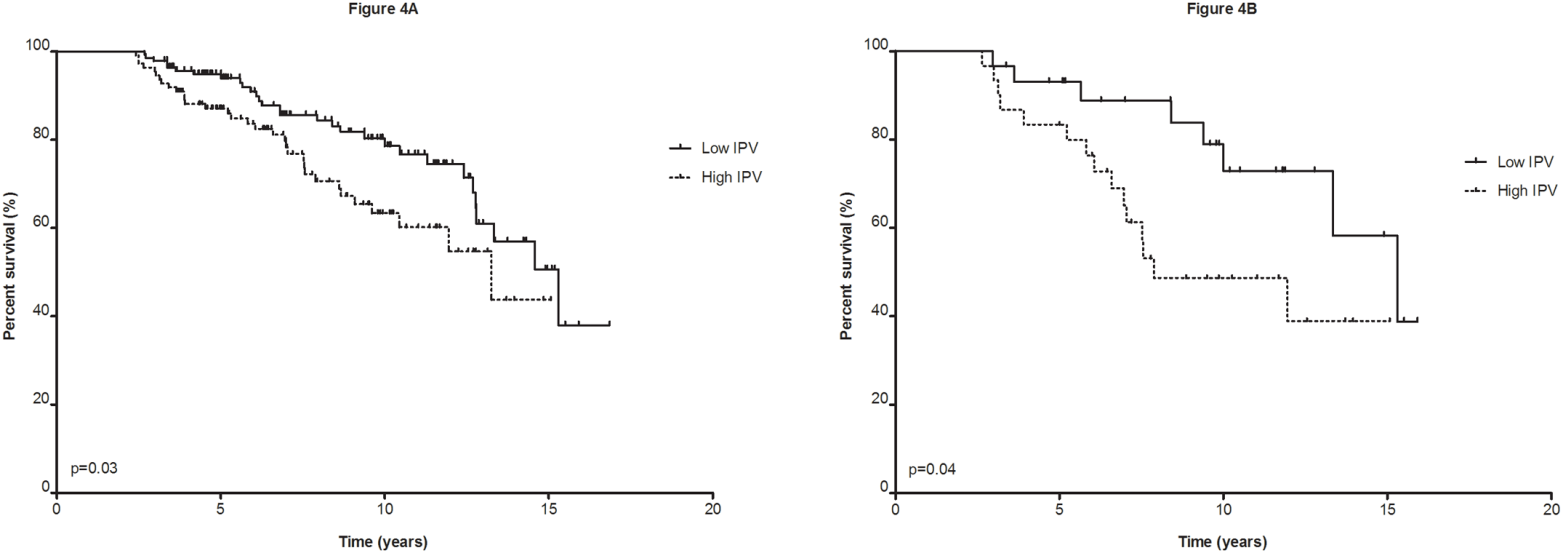
Allograft survival. (A) allograft survival based on ≤24% tacrolimus (Tac) intra-patient variability (IPV) vs. >24% Tac IPV of cases and controls combined, (B) Allograft survival of c-aABMR cases based on ≤24% Tac IPV vs. >24%.

To assess the association of intra-patient variability and Tac C_0_ with overall graft survival, the patients were divided dichotomously based on the cohort’s mean IPV and C_0_. The average IPV of the low IPV group (≤24%) was 18.5% and 30.4% for the high IPV group (>24%). Notably, a high IPV was significantly associated with inferior graft survival (13.3 years for ≤24% IPV vs. 11.2 for >24% IPV; log rank, p = 0.03)(Fig 4A). However, this association was only present for the cases. The cases with a low IPV showed an average survival of 13.1 years compared to only 9.9 years in cases with a high IPV (log rank, p = 0.04; Fig 4B). The controls had a survival of 13.4 years (≤24% IPV) versus 11.6 years (>24%)(log rank, p = 0.31).

**Fig 5:**
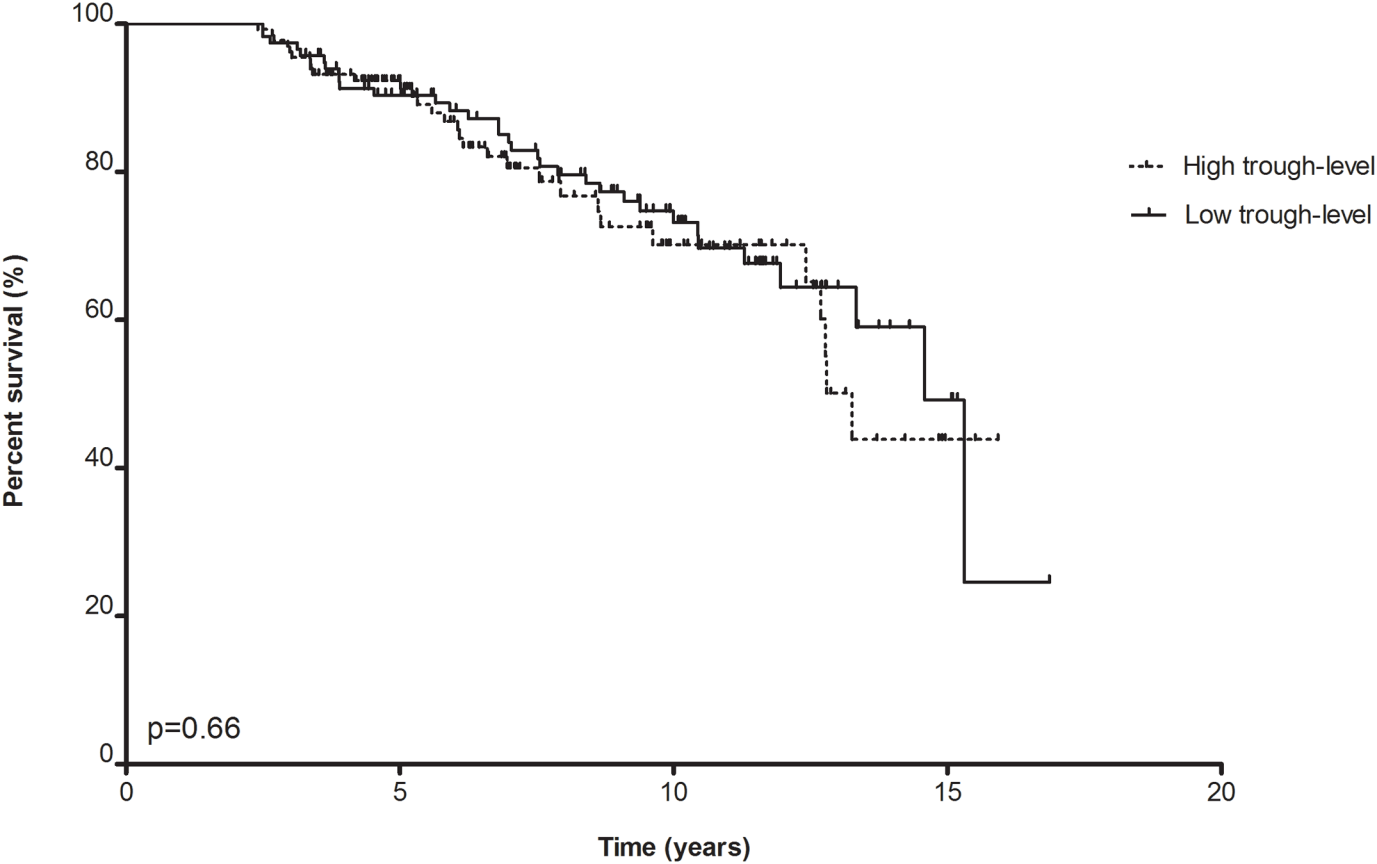
Allograft survival based on tacrolimus (Tac) predose concentrations (C_0_) ≤5.9 ng/mL vs. >5.9 ng/mL of c-aABMR patients (cases) and controls combined.

A similar analysis based on below or above average Tac C_0_ did not show any differences for either the case or control group (12.7 years for ≤5.9 ng/mL vs. 12.2 years; log rank, p = 0.66)(Fig 5).

## Discussion

In this retrospective case-control study we tested the hypothesis that a high IPV of tacrolimus predose concentrations is associated with a higher risk for developing chronic-active antibody mediated rejection of the kidney allograft. However, we found that in cases with c-aABMR the IPV for tacrolimus in the years prior to the diagnoses was similar to the IPV of matched controls.

Fluctuations in Tac C_0_ are very common in the first weeks or months after transplantation due to numerous causes such as drug-drug interactions, tapering of corticosteroid doses and/or changes in gastro-intestinal motility. Typically however, after a couple of weeks or months patients reach a more stable situation as the inter-occasion variability decreases. The differences in Tac C_0_ tend to get smaller over time. Fluctuations in Tac C_0_ in the maintenance phase after transplantation may therefor represent suboptimal patient-adherence. The calculated intra-patient variability of Tac C_0_ is considered a surrogate marker for non-adherence[9, 10, 23].

There are however several methods for the calculation of the fluctuation in Tac C_0_. One of which is the variance (σ^2^). The variance is used for the quantification of the intrapatient variability by showing the data distribution around the mean[13, 15]. Another useful method to determine the Tac IPV is by calculating the coefficient of variation (CV) [13, 24–28]. The CV assesses the degree of variation of Tac C_0_ measurements. We however, have chosen to use the mean absolute deviation (MAD) as a statistical measure for Tac IPV. Although the calculation of Tac IPV by means of MAD or CV are quite similar, the MAD is less susceptible to outliers. This is because the CV uses the squared deviation from the mean, whereas the MAD uses the absolute deviation[9, 11].

However, as stated above, the time period over which Tac C_0_ are collected, is of greater importance and should be taken into account when interpreting Tac IPV data. And even though a great variation has been reported in literature, on average Tac IPV is between 15% and 30% which is in line with our findings[11, 13, 24–28].

Borra *et al*. were the first to establish an association between high intra-patient variability in Tac C_0_ and the increased risk of developing graft loss[11]. Similar results have been published since[12–15, 29–31]. However, no evidence has been provided for a link between suboptimal Tac patient-adherence in the years after transplantation and the risk for late antibody mediated rejection of the renal allograft.

The current studies tend to rely on broad composite end-points consisting of a range of diagnoses such as late acute rejection, transplant glomerulopathy, graft failure and/or death with function[11–15]. It can be expected that considerably different results may be obtained once the composite endpoint is modified, as previously shown by the extension study of Borra *et al*[12]^2^. They demonstrated a substantially smaller effect size of Tac IPV on long-term allograft outcomes after adjustment of the composite endpoint. Furthermore a majority of these studies focus on IPV over a brief time span in the first year after transplantation while chronic humoral rejection is usually diagnosed at an average of 4–6 years after transplantation.

The remainder of studies on this subject define non-adherence as self-reported drug-noncompliance, clinical suspicions by the attending physician and/or repeated nonattendance at clinic visits or laboratory testing[3, 14]. This information is usually not present in most medical records except when the graft outcome was unfavorable which may create a biased interpretation of the results.

In this study, IPV in cases and matched controls was similar and could therefore not support the hypothesis that a high IPV is associated with an increased risk for the development of chronic active antibody mediated rejection. These findings are in line with previous results published by Halloran *et al*. where nonadherence was not significantly associated with late chronic active antibody mediated rejection[32]. Similarly, Vanhove *et al*. observed that high IPV of Tac predicted chronic allograft damage such as tubular atrophy and severe fibrosis but not inflammation or transplant glomerulopathy lesions[16].

Although cases and controls in our study had similar Tac C_0_ and renal function at starting point, the cases showed a significantly declining trend in both Tac C_0_ and renal function compared to the controls. The controls had a mean Tac C_0_ which varied around 6 ng/mL whereas the cases showed a gradual decline reaching Tac C_0_ of 5.4 ng/mL at endpoint. A similar development was visible in renal function where the controls showed an average decline in renal function of 1 ml/min/1.73m^2^ per year while the deterioration in graft function of the cases gradually increased to 10 ml/min/1.73m^2^ in the year prior to c-aABMR diagnosis. The decline in Tac C_0_ was significantly correlated to the decline in renal allograft function for the cases. Most likely, these observations reflect the physicians inclination to taper Tac dosage after witnessing a decline in allograft function, in an attempt to avoid presumed tacrolimus-related nephrotoxicity. Unfortunately, the entries made in the medical records were not sufficiently informative to support this conclusion with data.

Of interest is the significant association of an inferior graft survival for the c-aABMR patients with a high IPV versus those with a low IPV. This association was not found for the controls. This intriguing finding is in line with previous research suggesting poorer graft survival for patients with high IPV’s[11–15]. The explanation can only be speculative but it suggests that not the risk for development of c-aABMR is IPV-related but rather the subsequent severity of the ongoing humoral rejection. Adequate maintenance of Tac C_0_ seems to provide a better control of the chronic antibody mediated rejection which carries in general a poor prognosis for graft survival.

Our study does have several limitations. First, the causes of IPV cannot be determined due to the retrospective nature of the study. Even though multiple factors are known to contribute to a high IPV, nonadherence is considered a dominant cause[10, 16]. Secondly, external validation might not be possible while it is a single center study and may not reflect the transplant population of other centers and their immunosuppressive regimen. However, the Tac C_0_ were in range with what is considered common practice (6.1 ng/mL and 5.8 ng/mL).

In conclusion, we demonstrated that a high intra-patient variability of tacrolimus long-term after kidney transplantation is not associated with the occurrence of chronic antibody mediated rejection but unfavorably affects graft survival in patients with c-aABMR.

## Supporting information

S1 File The data base used in this study. (SAV)
